# Morphometric and Nanomechanical Screening of Peripheral Blood Cells with Atomic Force Microscopy for Label-Free Assessment of Alzheimer’s Disease, Parkinson’s Disease, and Amyotrophic Lateral Sclerosis

**DOI:** 10.3390/ijms241814296

**Published:** 2023-09-19

**Authors:** Stefka G. Taneva, Svetla Todinova, Tonya Andreeva

**Affiliations:** 1 Institute of Biophysics and Biomedical Engineering, Bulgarian Academy of Sciences, “Acad. G. Bontchev” Str. 21, 1113 Sofia, Bulgaria; todinova@abv.bg (S.T.); tonya.andreeva@reutlingen-university.de (T.A.); 2 Faculty of Life Sciences, Reutlingen University, Alteburgstraße 150, D-72762 Reutlingen, Germany

**Keywords:** platelets, red blood cells, cell morphology, cell nanomechanics, atomic force microscopy, biomarkers, neurodegenerative disorders

## Abstract

Neurodegenerative disorders (NDDs) are complex, multifactorial disorders with significant social and economic impact in today’s society. NDDs are predicted to become the second-most common cause of death in the next few decades due to an increase in life expectancy but also to a lack of early diagnosis and mainly symptomatic treatment. Despite recent advances in diagnostic and therapeutic methods, there are yet no reliable biomarkers identifying the complex pathways contributing to these pathologies. The development of new approaches for early diagnosis and new therapies, together with the identification of non-invasive and more cost-effective diagnostic biomarkers, is one of the main trends in NDD biomedical research. Here we summarize data on peripheral biomarkers, biofluids (cerebrospinal fluid and blood plasma), and peripheral blood cells (platelets (PLTs) and red blood cells (RBCs)), reported so far for the three most common NDDs—Alzheimer’s disease (AD), Parkinson’s disease (PD), and amyotrophic lateral sclerosis (ALS). PLTs and RBCs, beyond their primary physiological functions, are increasingly recognized as valuable sources of biomarkers for NDDs. Special attention is given to the morphological and nanomechanical signatures of PLTs and RBCs as biophysical markers for the three pathologies. Modifications of the surface nanostructure and morphometric and nanomechanical signatures of PLTs and RBCs from patients with AD, PD, and ALS have been revealed by atomic force microscopy (AFM). AFM is currently experiencing rapid and widespread adoption in biomedicine and clinical medicine, in particular for early diagnostics of various medical conditions. AFM is a unique instrument without an analog, allowing the generation of three-dimensional cell images with extremely high spatial resolution at near-atomic scale, which are complemented by insights into the mechanical properties of cells and subcellular structures. Data demonstrate that AFM can distinguish between the three pathologies and the normal, healthy state. The specific PLT and RBC signatures can serve as biomarkers in combination with the currently used diagnostic tools. We highlight the strong correlation of the morphological and nanomechanical signatures between RBCs and PLTs in PD, ALS, and AD.

## 1. Introduction

The increase in human life expectancy is contributing to an increase in the number of patients with neurodegenerative diseases (NDDs)—Alzheimer’s disease (AD), Parkinson’s disease (PD), and amyotrophic lateral sclerosis (ALS) being the most common ones. NDDs are age-dependent pathologies [1] that share common dysfunctions in fundamental cellular processes, such as mitochondrial blood-brain barrier dysfunction [2,3,4], oxidative stress and generation of reactive oxygen species [5,6,7], dysregulation of calcium homeostasis [8,9,10], and are associated with progressive degeneration and/or loss of neurons in the central and peripheral nervous systems, movement problems, and/or memory impairment. Besides age, sex dimorphisms play an important role in the development and progression of AD, PD, and ALS [11]. In most cases, the causes of NDDs are idiopathic. The presence of environmental toxins, genetic predisposition, and the processes of oxidative stress and inflammation associated with the aging of the body play a role in the etiology of these conditions [1]. The genetic contribution was found to be higher in AD, Huntington’s disease, and brain degeneration and lower in PD, a motor neuron disease, and prions [12]. One of the current theories about the occurrence of NDDs is that it is largely related to systemic inflammation [13,14]. The immune cells that underlie the neuro-inflammatory response are involved in the clearance of accumulated pathological proteins and ensure the survival of neighboring neurons and the maintenance of brain homeostasis. These cells, however, can release molecules that promote oxidative stress and damage to surrounding neurons and abnormally remove healthy cells from the brain [13,14,15]. Recent studies have also shown that there may be a correlation between genetic and environmental agents, including exposure to heavy metals, pesticides, dietary habits, stress, and other factors such as inflammatory cytokines, leading to modulation of the normal functioning of the central nervous system and increased neuronal sensitivity to oxidative stress or apoptosis [16].

Abnormal accumulation of insoluble, toxic aggregates from misfolded specific proteins (β-amyloid peptide (Aβ), tau-protein (tau), and α-synuclein (α-syn)) in the brain and in peripheral body fluids, blood cells, and tissues [17,18,19,20,21] is a common mechanism of NDDs, also called “protein misfolding disorders” [22,23,24]. Recent findings proved that misfolded proteins cannot only self-assemble [25], but also interact with each other or with other “pathological proteins”, forming toxic heteroaggregates present in the brains and peripheral blood cells of patients [18,26,27].

Despite the advances in diagnostic and therapeutic methods, the treatments applied only alleviate the symptoms or slow the disease’s progression. Furthermore, motor and/or cognitive deficits usually appear at a relatively advanced stage of neurodegeneration, and hence neuronal loss in the substantia nigra and dopaminergic deficiency in PD patients, and accumulation of protein plaques and tangles resulting in neuronal dysfunction and cell death in AD patients, appeared before the clinical diagnosis. This is because of the lack of reliable biomarkers that allow early diagnosis of NDDs. Therefore, the development of new approaches for cheaper and faster diagnostics, respectively, for the detection of novel reliable, more cost-effective, and readily accessible diagnostic biomarkers and the establishment of new therapies for these diseases is of utmost importance [19,27,28]. The pathological features of these diseases allow such markers to be identified in peripheral blood cells, peripheral fluids, and tissues.

## 2. Current Biomarkers for the Diagnosis of Alzheimer’s Disease, Parkinson’s Disease, and Amyotrophic Lateral Sclerosis

Currently, the diagnosis of the majority of NDDs is based on clinical examination combined with a number of laboratory methods: liquid biopsy, biophysical, biochemical, genetic, imaging, “omics” techniques, machine learning, etc. [19,29,30,31,32,33,34,35].

Brain imaging techniques, such as magnetic resonance imaging (MRI) [36], diffusion and advanced diffusion MRI, and alternative imaging approaches (diffusion tensor imaging, neurite orientation dispersion and density imaging, free-water imaging, etc.), are powerful noninvasive tools for the detection of brain biomarkers and microstructural characteristics of the brain [37,38]. Positron emission tomography (PET) is another promising tool to identify abnormal brain metabolism (^18^F-FDG PET) [39] and to detect amyloid deposition (Amyloid PET) [40,41,42,43] and disease staging with amyloid and tau PET [44]. These techniques, however, are expensive and not routine clinical practice. “Omics” technologies are also effective tools for the detection of molecular biomarkers. Proteomics and metabolomics can detect proteins and neurotransmitter metabolites involved in NDDs [45,46], and create a characteristic profile of specific biomarkers for certain pathologies or for several pathologies sharing similar characteristics [47,48].

Considerable efforts are ongoing to identify fluid biomarkers, specific proteins/peptides, and miRNAs in body fluids, which are readily detected in both cerebrospinal fluid (CSF) and blood plasma [19,48,49,50]. CSF is one of the most important sources of biomarkers because it directly reflects all biochemical changes in the brain [51], which are also sensed in blood plasma. Both CSF and blood plasma were screened for the presence of NDD biomarkers and potential differential diagnoses [30,52,53,54]. Among the fluid markers for the detection and monitoring of the preclinical and clinical stages of NDDs, the most established ones are Aβ42, total tau-protein (t-tau), phosphorylated tau-protein (p-tau181), and neurofilament light (NfL) in AD [55], α-syn in PD [56,57,58,59,60,61] and clusterin [62], creatinine, albumin, transactive response DNA binding protein 43 kDa (TDP-43) in ALS [63,64,65], as well as RNA biomarkers [66]. While CSF sampling is an invasive procedure, blood plasma is more accessible and less invasive; however, the biomarkers in plasma are present in low concentrations and are difficult to detect [33]. In recent years, high-sensitivity techniques, such as mass spectrometry [67] and digital ELISA, that allow the detection of compounds at low concentrations have been developed [68]. Plasma biomarkers (Aβ42, Aβ40, α-syn, p-tau181, t-tau, NfL, and TDP-43) were also identified by means of single-molecule array technology (SIMOA) [69].

### 2.1. Fluid Biomarkers in Alzheimer’s Disease

The combination of several plasma biomarkers has demonstrated improved accuracy for AD diagnosis compared to individual ones, which also showed good accuracy [70,71,72,73]. It has been established that CSF p-tau levels increase while CSF Aβ levels decrease in AD [74,75,76]; the plasma ratio Aβ42/Aβ40 and p-tau181 protein can distinguish between AD and a healthy state [77,78,79,80]. The plasma Aβ42/Aβ40 ratio significantly correlates with Aβ accumulation detected by mass spectrometry assay and by PET scans; the latter also predicted AD progression [81]. Some authors postulated the use of p-tau181 as a marker for disease progression [82,83]. Additionally, hyperphosphorylated tau isoforms could be helpful for distinguishing AD with greater accuracy, especially at early disease stages [84,85].

Analysis of the combination of some potential plasma biomarkers (p-tau181, t-tau, Aβ42, Aβ40, NfL, TDP-43, t-tau/Aβ42, and Aβ42/Aβ40 ratio) in AD by means of SIMOA revealed the range of normal and pathological plasma concentrations for each marker and the correlations of their plasma levels with the corresponding CSF levels ([69] and references therein). No correlation was obtained between CSF levels of Aβ42 and t-tau protein and their plasma levels. A positive correlation was determined between the levels of both p-tau181 and NfL in plasma and CSF; p-tau181 in plasma also correlated with t-tau and the t-tau/Aβ42 ratio in CSF. Inversely, plasma Aβ42/Aβ40 ratio correlated negatively with CSF t-tau, CSF p-tau181, and CSF t-tau/Aβ42 ratio.

Recent data demonstrated that neurodegeneration of any etiology is reflected in CSF and plasma levels of t-tau, p-tau231, and p-tau217, which were suggested as the most promising blood biomarkers, reaching abnormal levels at early cerebral Aβ changes [84,86,87,88]. Importantly, plasma p-tau231 combined with the Aβ42/40 plasma ratio manifested the largest change in patients’ cohorts with low Aβ levels [88].

### 2.2. Fluid Biomarkers in Parkinson’s Disease

Although there are some contradictory results, α-syn protein is considered the most promising biomarker for PD; abnormal CSF and plasma levels of oligomeric α-syn (o-α-syn) were reported to correlate with brain abnormalities in PD patients [89,90,91]. Besides toxic α-syn homoaggregates, pathological heteroaggregates of α-syn and the classical AD biomarkers, Aβ and tau proteins (α-syn/Aβ/tau) are also related to the occurrence of PD [12,92,93,94,95,96]. A recent study of variously sized α-syn aggregates separated from a heterogeneous mixture by density centrifugation proved that the cytotoxic properties correlated with the aggregate size, with the small non-fibrillar aggregates being more toxic than the large ones [97]. Importantly, soluble aggregates extracted from post-mortem PD brains had a similar size and structure as the smaller, more toxic aggregates separated from a mixture of α-syn aggregates [97].

Combination of the ratio o-α-syn/total α-syn (t-α-syn) and the age of patients, CSF t-α-syn and Aβ42, t-tau and p-tau, and β-glucocerebrosidase activity can differentiate PD patients from healthy subjects [98,99], while CSF p-α-syn/t-α-syn, CSF Aβ42, p-tau, and, in particular, CSF NfL levels are associated with cognitive performance in early-stage PD patients. A greater diagnostic accuracy was reached by combining oligomeric t-α-syn and Aβ42/tau ratios [99]. Plasma levels of α-syn, Aβ-40, and t-tau are also recognized as predictive markers for cognitive decline [61,100,101]. Several inherited, familial mutations have been found to correlate with elevated PD risk and to perturb the α-syn structure [102]. The levels of t-α-syn, proteinase K-resistant (PKres) α-syn, phosphoserine 129 α-syn and oxidized α-syn were suggested as complex biomarkers for PD [103].

### 2.3. Fluid Biomarkers in Amyotrophic Lateral Sclerosis

There are still no clinically validated, reliable, and specific markers for ALS [104]. Combining clinical trials, MRI analysis applying pattern-recognition algorithms, and machine learning is expected to provide an earlier diagnosis of ALS and a prediction of the disease course [105]. Recently, biomarkers in CSF have been identified, including the levels of TDP-43 protein [106,107], chemokines [108], and NfL [109,110]. The ALS-linked genetic mutations encoding TDP-43 and copper-zinc superoxide dismutase 1 (SOD1) proteins have demonstrated distinct metabolic phenotypes. TDP-43 leads to a decrease in carnitine and an increase in pyruvate and fatty acids [111,112], and SOD1 leads to a drop in arginine, lysine, ornithine, serine, threonine, and pyroglutamic acid [113] in ALS patients with these mutations. TDP-43 [114] and SOD1 [115] are some of the proteins implicated in both familial and sporadic ALS, although it is still unclear whether they are a cause of the pathology or a symptom. As discussed in [107,116], TDP-43 protein forms toxic aggregates in the cytoplasm of motor neurons, which are detected in the majority (97%) of ALS patients; however, its role as a biomarker is still a matter of debate [107].

A large number of studies reported elevated levels of inflammatory markers—cytokines, TNFα, IL-1β, IL-6, IL-8, TNF receptor 1, and vascular endothelial growth factor (VEGF)—in ALS serum and plasma; however, the inflammatory markers show no specificity for ALS diagnosis and progression ([116,117] and references therein). Also, C-reactive protein (CRP), an inflammation marker, is elevated in the serum of preclinical ALS patients and correlates with the rapid progression of the disease [118].

The cytoskeletal protein NfL and its phosphorylated form, pNfH, are increasingly recognized as the most promising diagnostic biomarkers for ALS [110,119,120], which are correlated to rapid progression and a worse prognosis of the disease [121,122]. Accumulation of aberrant NfL was detected in ALS patients with familial and sporadic forms of the pathology [123]. Higher levels of both CSF and plasma NfL were detected in the early stages of ALS and were found to correlate with shorter survival [124,125]. Plasma NfL can differentiate and mimic ALS phenotypes [126]. It should be noted that the NfL level in CSF is also elevated in AD [127] and PD [128], as discussed above, as well as in other neurological disorders. Multivariate analysis of CSF proteins has shown that NfL and t-tau proteins were correlated with ALS progression, and plasma NfL was correlated with the ALS diagnostic grade [129]. A negative correlation between CSF NfL and TDP-43 was revealed by mutual biomarker analysis [129].

Furthermore, human serum albumin and creatinine were suggested as independent markers in ALS and also as indicators of the disease’s severity [130]. Albumin in ALS patients was reported to correlate with inflammatory markers and creatinine with a marker of muscle mass [63]. Increasing levels of CRP and glucose were detected during the very fast progression of ALS [130].

Additionally, Colletti et al. [131] found that Aβ42 may be involved in the pathogenesis of ALS and that the Aβ42/Aβ40 ratio may serve as a prognostic biomarker.

Plasma and CSF levels of lipids associated with ALS pathological pathways have been found to correlate with disease progression [132,133]. Increased incidence of ALS was related to serum low-density and high-density lipoprotein cholesterol (LDLC and HDLC), apolipoprotein B, and other lipids [134,135]. Cholesterol and LDL/HDL levels were also associated with ALS development [136].

## 3. Platelets and Red Blood Cells in Alzheimer’s Disease, Parkinson’s Disease, and Amyotrophic Lateral Sclerosis

How platelets (PLTs) and red blood cells (erythrocytes, RBCs) are affected in NDDs and what their role is in the development of these diseases is not fully understood and remains to be studied and elucidated. Changes in the number and function of both types of blood cells have been observed in various NDDs, including AD, PD, and ALS. However, these changes are not specific to any particular neurodegenerative disease and may also occur in other diseases. In this review, we seek to summarize the advances in the attempt to explore specific changes in the morphological and mechanical properties of PLTs and RBCs, as well as AFM as medical diagnostic tools to detect NNDs and differentiate between them.

### 3.1. Platelets

PLTs are small, anucleate multifunctional cells with a primary role in hemostasis and thrombosis [137], but they are also involved in inflammatory processes that contribute to different pathologies, like cardiovascular disease and cancer, and are risk factors for neurodegenerative diseases and their progression [138,139,140,141]. Various neurodegenerative pathological conditions, including PD, AD, and ALS, were linked to platelet dysfunction [142], activation, and aggregation [143,144,145]. Furthermore, oxidative and physiological stress induce structural and functional alterations and PLT activation in AD, ALS, and PD [52,143,144,145,146]. The PLT cytoskeleton is a dynamic, complex protein network that plays a key role in platelet function [147]. Upon activation, the PLT cytoskeleton and hence the cell membrane morphology undergoes significant reorganization [140,148,149,150,151].

In AD, a reduced PLT count was found, which was associated with a deterioration of cognitive functions [146,152,153,154], while PD patients had an increased number of PLTs [141].

Furthermore, the expression of some proteins connected to the pathogenesis of AD, such as the transmembrane amyloid precursor protein (APP) [155,156] and tau protein [157], was enhanced; likewise, reactive oxygen species accumulation, which was associated with PLT dysfunction [146]. The increased level of APP [158] and production of Aβ peptides in AD stimulated PLT activation and aggregation [159,160,161], and in turn, PLT activation might induce changes in cell membrane fluidity [162]. Changes in PLT activation and aggregation state, membrane ultrastructural modifications, mitochondrial abnormalities, and dysfunction have also been found in ALS [52,143,146,163,164], and PD [144] patients.

Besides, overexpression of platelet α-syn in PD was also associated with mitochondrial dysfunction and oxidative stress. [159,165,166,167]. Furthermore, PLT serotonin levels were found to be lower in patients with ALS and to positively correlate with patients’ survival [168]. Likewise, the serotonin level was diminished in AD PLTs, while the AD plasma serotonin level was increased [169]. Evidence was provided for direct interaction between PLTs and specific glycolipid structures present in the lipid raft domains of neuronal cells [170,171].

Similar to neurons, which contain intercellular storage compartments for neurotransmitters, neuropeptides, and neurohormones [172], PLTs have the ability to store and release neurotransmitters, intercellular signaling molecules, and other bioactive molecules in α- and dense granules, which is essential for maintaining brain function and intercellular communication [173]. The α-granules store proteins such as fibrinogen, coagulation and growth factors, adhesive molecules, cytokines, and chemokines [174], while dense granules are a storage pool for small molecules such as ADP, ATP, polyphosphate, serotonin, and calcium [175]. The α- and dense-platelet granules resemble the large dense-core and the small dense-core synaptic vesicles of neurons, respectively [175]. Intercellular communication can also be conducted by the release of extracellular vesicles, exosomes, and microparticles that contain bioactive molecules and microRNAs [176]. The exocytosis in PLTs and neurons is activated by an increase in the internal calcium concentration [141], leading to the activation of the secretory machinery.

An increased extracellular concentration of glutamate, one of the major excitatory neurotransmitters, and a decline in glutamate uptake have been reported to mediate glutamate excitotoxicity in AD and PD, which is likely related to the abnormal aggregation of amyloid peptides (Aβ and α-syn) [177,178]. The ALS glutamate excitotoxicity was attributed to an atypical increase in glutamine synthetase in platelets [179,180].

Moreover, when the blood-brain barrier is compromised, PLTs can communicate with neuronal cells and, once activated, release bioactive molecules and neurotransmitters, thus playing a pro-inflammatory role [181,182]. Inhibition of platelet activation and aggregation is used for the development of anti-platelet therapy to reduce and treat cardiovascular disease and is also expected to be applied to the treatment of neurodegenerative disorders ([141] and references therein). On the other hand, the therapeutic potential of healthy platelets (platelet lysate and platelet-rich plasma) is also a challenge.

### 3.2. Red Blood Cells

Human RBCs are deprived not only of the nucleus but also of all subcellular organelles, including mitochondria [183]. Nevertheless, all tissues depend on the functioning of RBCs, especially neurons, which use 20% of the total oxygen consumed. In structural terms, the red blood cell is maintained by a membrane cytoskeleton with a 2D six-fold structure consisting of a dense network of spectrin tetramers linked to the phospholipid bilayer by “binding complexes” and ankyrins [184,185,186,187,188]. The binding complexes contain the Band 3 protein, located in the lipid bilayer as a dimer or tetramer, bound to ankyrin, thereby making links to the cytoskeleton via the spectrin network [189]. Glycophorins and Band 3 proteins are associated with the cytoskeleton and have an important role in the maintenance of RBCs’ shape and mechanical properties [190]. Band 3 protein also plays a major role in cell metabolism and in the exchange of oxygen between hemoglobin (Hb) and tissues [186,189,191]. The unbound parts of the membrane cytoskeleton are flexible and allow a dynamic change of the RBC’s shape and significant deformation without damage during the passage of the cells into the bloodstream [192]. A change in a single component of the membrane cytoskeleton can lead to a modification of the whole structure, which would impair the cells’ function and the oxygen transport mechanism.

RBCs may be thought of as biochemical machines that can be structured, aged, and removed from the bloodstream (along their 100-day lifespan) when their function is impaired. Cellular aging is accompanied by a strong reduction in cell volume and Hb content, but the mechanisms of these events are not elucidated [192,193,194].

The unique biconcave shape and the reversible deformations, critical for the function of RBCs, are maintained by the lipid bilayer plasma membrane [186,187]. Importantly, the remarkable ability of RBCs to deform is critical for their primary function, oxygen transport through the bloodstream, and can change significantly under different pathophysiological conditions, which might be useful for the differential diagnosis of various diseases [195]. Along the aging process, RBCs undergo morphological transformations from the well-known smooth and biconcave forms at rest and under normal physiological conditions to different atypical morphological types [196,197,198,199,200].

The mature RBCs are involved in interactions with other cells in the peripheral blood, like endothelial cells, platelets, macrophages, and bacteria [183], which are mediated by proteins such as fibrinogen or immunoglobulins. Furthermore, RBCs release extracellular vesicles, endosome-derived exosomes, and microvesicles—membranous extracellular structures containing biomarkers and microRNAs but not DNA, suggesting their involvement in cell-cell interactions, thrombosis, systemic inflammation, or cell adhesion [188,201]. During their lifespan, RBCs also shed hemoglobin-containing vesicles in the circulation, an event supposed to control the changes in the cells’ physical features during their maturation [202].

Decreased RBC counts have been shown to occur in AD patients and may be associated with an increased risk of dementia and cognitive impairment [203]. The degree of reduction in the levels of RBCs, Hb, and hematocrit compared to healthy individuals depends on the disease stage and is more significant in late-stage than in early-stage PD [204]. The severely reduced Hb concentration is thought to be related to disease duration and peripheral iron metabolism.

Like in peripheral fluids and PLTs, significant amounts of Aβ are detected in RBCs, suggesting that plasma Aβ peptides can interact with RBCs [165]. Besides, the concentrations of Aβ42 and Aβ40 were described to increase with age and to be significantly higher in RBCs from older than from young healthy individuals [26]. The higher Aβ level induces oxidative injury and impairs RBC capacity [26,205,206].

Recent studies have shown that RBCs from patients with AD are linked to amyloid peptides [20], suggesting a pathogenic role for RBC-amyloid peptide complexes. The binding interaction between RBCs and Aβ in the blood stream leads to oxidative stress and the generation of reactive oxygen radicals in erythrocytes [207], thus disrupting their oxygen delivery capacity and facilitating disease development. The Aβ-erythrocyte complex induces changes in the cells’ morphology and their adhesion to the endothelium, thereby affecting endothelial viability and functionality [208].

Although there are some contradictory results on the total α-syn levels in RBCs [89], as well as α-syn in plasma/sera [91], higher levels of o-α-syn were found in PD patients compared to controls [209,210,211]. It is supposed that the source of α-syn might be intact or lysed RBCs that entered the cerebrospinal fluid [165] or that α-syn is secreted by RBCs in the form of extracellular vesicles that can cross the blood-brain barrier [212]. Recent experimental evidence also demonstrated a higher level of p-tau in PD RBCs [209] and a correlation of the RBCs’ t-tau protein concentrations with cognitive deficits in newly diagnosed patients [213]. In addition, the interaction of α-syn with Aβ42 and tau protein in PD RBCs has also been reported [209]. The elevated level of α-syn-Aβ42 heteroaggregates was particularly found to correlate with disease severity and motor deficits in PD patients [209].

Furthermore, as reported by Baldacci et al. [25], α-syn/Aβ and α-syn/tau heterodimers in AD RBCs can differentiate between AD patients and healthy subjects, whereas RBC α-syn concentrations alone cannot. The secretion of damaging molecules by RBCs is thought to contribute to the development of ALS. Indeed, a correlation was found between the progression of the disease and increased activity of acetylcholine esterase, increased erythrocyte deformability, and reduced flow of nitric oxide from RBCs [214].

## 4. Atomic Force Microscopy: Morphometric and Nanomechanical Parameters in Neurodegenerative Pathologies

### 4.1. Atomic Force Microscopy as a Diagnostic Tool

**Figure 1:**
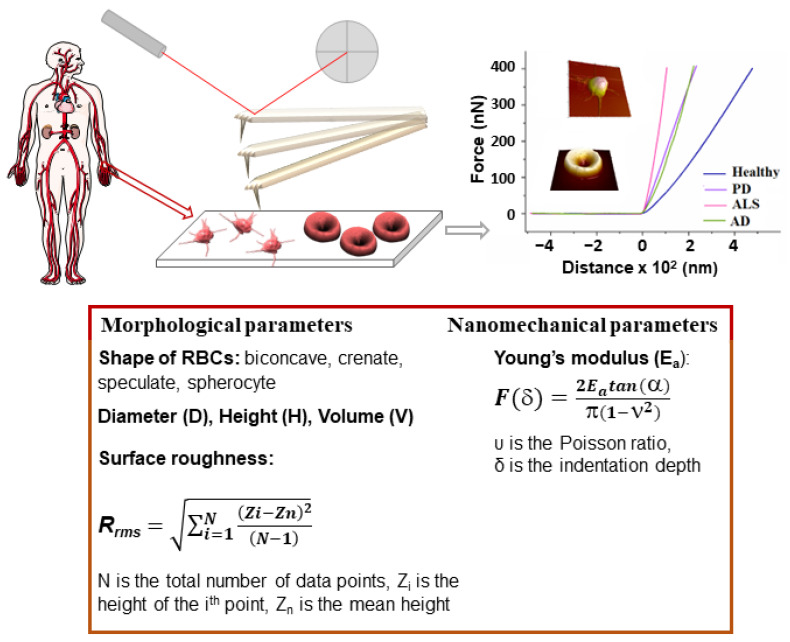
Schematic of the AFM-based approach for diagnosis of NDDs: platelet-reach plasma or RBCs isolated from venous blood samples derived from patients and healthy volunteers; fixation of PLTs and preparation of smears of RBCs on a microscope glass slide; AFM scanning; morphometric and nanomechanical parameters evaluated from the 3D images of PLTs and RBCs and force-distance curves, respectively. The morphometric and nanomechanical parameters obtained by AFM scans are listed in the box.

Atomic force microscopy (AFM) is a versatile tool to investigate the topography and properties of surfaces, as well as the properties of single molecules and intermolecular interaction forces. AFM generates high-resolution images of the surface of biomolecules, membranes, cells, and tissues, and can also probe their mechanical, chemical, electrostatic, and biological properties [215]. The AFM basic principles, the modes of imaging of biointerfaces, molecular and force spectroscopy, and the advantages and limitations of AFM-related techniques have been reviewed in [215,216]. The main capability of AFM is to detect the weak forces acting between a very sharp tip (called a probe) and the sample under examination. The probe is attached to a flexible cantilever, which deforms as a result of the forces of attraction and repulsion between the tip and the surface (Figure 1).

This deformation is reflected onto the position of a laser beam on a position-sensitive detector, thus producing a three-dimensional image (topography) of the sample surface with nanometer resolution. In addition to imaging, due to the fact that AFM is able to measure extremely weak forces in the range of intermolecular interactions, it has been applied to assess the nanomechanical properties of objects by nanoindentation, a process providing various parameters, such as the Young’s modulus (stiffness, E_a_) (Figure 1), which is widely used in cell biology.

Due to its excellent visualization and measurement capabilities, combined with unprecedented precision and spatial resolution, AFM has proven to be a valuable tool for studying biological samples like proteins, cells, bacteria, and viruses ([217] and references therein). This information is important to deepen our understanding of protein functions and disease mechanisms [218]. Recently, a comprehensive review summarized data on the application of AFM in membrane biophysics, especially in the study of model membranes, lipid-protein interactions, and the formation of Aβ42 fibrils ([219] and references therein). AFM can also be used to image individual proteins and analyze their structural characteristics. Another distinctive aspect of AFM that qualifies it as a potential diagnostic tool is the fact that it requires minimal sample manipulation and allows examination of one object in its native environment. It has been established as a valuable platform to study the morphological and mechanical characteristics of living biological objects, for example, for the identification and visualization of orthopoxviruses and orthopoxvirus particles in clinical suspensions [220,221,222].

In medicine, AFM has various applications; one of them is the study of the mechanical properties of cells, tissues, and organs. By using AFM, scientists can determine the elasticity, adhesion, stiffness, and other mechanical parameters of biological samples. This can help characterize disease states and study the effect of drugs at the cellular level [221,222]. AFM capabilities have been exploited to distinguish between cancerous and normal cells in both morphological [223] and nanomechanical signatures [224,225]. AFM imaging demonstrated 94% diagnostic accuracy in the detection of specific features on the surface of bladder cancer cells found in patients’ urine [226].

In addition, AFM can be combined with other imaging techniques, such as optical microscopy, to provide a more comprehensive characterization of biological samples. This combination allows both morphological and mechanical information to be obtained, providing a more complete picture of the object under investigation [222].

Overall, the application of AFM in medicine helped to increase the understanding of biological systems and gain new insights into diseases and their treatment options.

### 4.2. AFM Studies of Platelets and Red Blood Cells in Healthy and Pathological Conditions

3D images of RBCs obtained using AFM were first reported in 1990 [227,228]. Later on, the technique was applied to compare the overall morphological and surface details of normal and pathological RBCs in smears fixed in glutaraldehyde [229].

In the blood of a healthy person, the majority of RBCs are characterized by a typical biconcave shape and a regular ultrastructure, and only a very small fraction are cells with irregular morphology [197,230,231]. Important insights into the surface roughness parameter (R_rms_) of RBCs as a measure of the cell-membrane skeleton integrity and functional status have been given by Girasole’s research group [196,232,233]. The authors found that R_rms_ is independent of cell shape and has a constant value over the entire surface of a single cell and within a particular sample, whether healthy or pathological. While Kamruzzahan et al. [234] proposed imaging the cells with a gentler tapping mode AFM, Girasole et al. [233] reported that the tapping mode provided undervalued average surface roughness and suggested using contact mode for the investigation of the RBC membrane skeleton and its modifications.

AFM revealed significant differences between healthy and a variety of pathological cells [234,235,236]. For example, AFM images revealed characteristic circular-shaped holes on the surface of RBCs from systemic lupus erythematosus patients [234]. RBC morphological parameters (width, length, length to width ratio, valley, peak, valley-to-peak, and surface fluctuation) have been shown to distinguish multiple myeloma (MM) and Waldenström macroglobulinemia from healthy cells and to be useful to follow the effect of disease treatment [237,238]. RBCs from MM patients were extremely deformed and had a macrocytic and canthocytic shape compared with the biconcave healthy shape [237]. Dramatic deformations of the shape and membrane surface of RBCs have been associated with iron deficiency anemia and thalassemia; moreover, the AFM parameters made it possible to distinguish iron deficiency anemia and thalassemia [230]. Increased numbers of abnormally shaped red cells and acanthocytes were detected in subjects with Panthothenate kinase-associated neurodegeneration (PKAN), a hereditary neurodegenerative disorder [239]. The morphological abnormalities indicated perturbed cytoskeleton and lipid bilayer organization and altered microparticle formation [239]. Nanomechanical, spectroscopic, and lipidomic studies revealed a decreased rigidity in the interfacial region of the RBC membranes of obese subjects related to a changed lipid composition: an increase in the cholesterol/phospholipid mole ratio of some ω-6 fatty acids and a decrease in sphingomyelin contents as compared to healthy subjects [240]. Exploring AFM, relationships have been established between cytoskeleton destruction, disturbance of the membrane nanostructure, and the morphology of RBCs caused by many physical and biochemical factors [241].

It became evident that various disease conditions are associated with changes in the PLT’s mean size and, correspondingly, in the PLT’s volume, which is also related to the PLT’s function. A multitude of medical conditions are associated with high platelet volume, including cancer, diabetes, cardiovascular disease, preeclampsia, Crohn’s disease, hyperthyroidism, immune thrombocytopenia, myeloproliferative disease, vitamin B12, vitamin D, or folate deficiency, macrothrombocytopenia (giant platelet disorders), etc. [242]. For example, it is well documented that in patients with immune thrombocytopenia, the PLT count is decreased, as is the PLT life span, but the existing PLTs become larger in size in order to compensate for the reduced count [243]. The PLT diameter in such patients was found to be 1.6 times higher than the mean PLT diameter in healthy individuals [243]. A clear example of how platelet size can serve as a prognostic marker is that patients with cardiovascular disease and increased platelet volume show a higher risk of thromboembolic complications and a fatal outcome [244]. Analysis of a large pool of data for the mean volume of PLTs from healthy individuals and patients with acute myocardial infarction showed ca. 8% consistent increase in the volume [244]. On the other hand, there are medical conditions associated with a decrease in PLT volume, such as tuberculosis, ulcerative colitis, systemic lupus erythematosus (SLE) in adults, and different neoplastic diseases [242].

Therefore, changes in the PLT’s size can be an important marker for disease state and may also be used to monitor disease progression or response to treatment. However, it is important to note that changes in PLT size are not specific and must be considered along with other clinical factors to make a diagnosis for a certain disease.

### 4.3. Morphometric and Nanomechanical Parameters of Red Blood Cells and Platelets in Neurodegenerative Disorders

**Figure 2:**
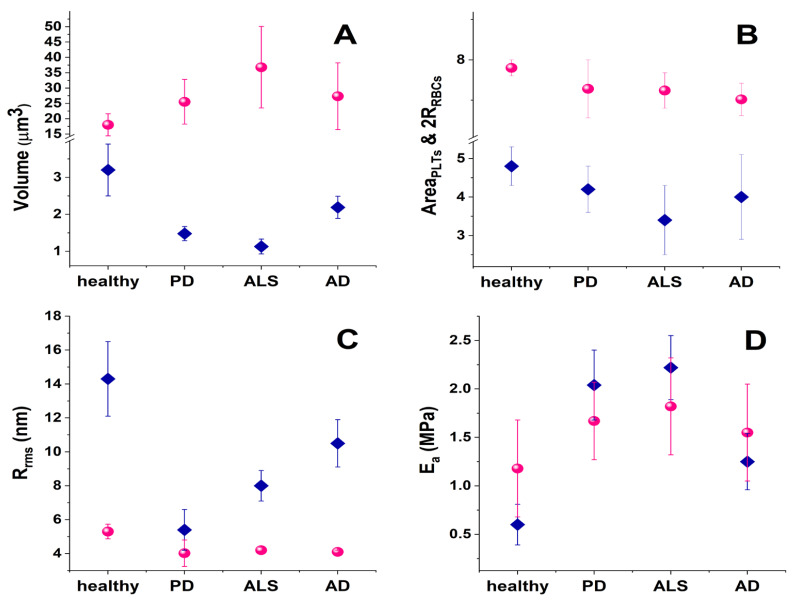
Mean morphological and mechanical parameters of PLTs (pink spheres) and RBCs (blue diamonds) in blood from healthy subjects and in NDD pathologies (**A**) cell volume; (**B**) cell spreading area; (**C**) cell membrane root mean square roughness, R_rms_; (**D**) cell membrane Young’s modulus, Ea.

Studies of the morphometric and mechanical parameters of PLTs and RBCs in NDDs are scarce so far. Ultrastructural modifications in ALS PLTs and platelets’ mitochondria, pseudopodia formation, and platelet activation have been revealed by transmission electron microscopy [245]. Our investigations revealed a considerable shrinking of about 64.7% of the PLT volume for patients with ALS and about 53.7% and 31.6% for those suffering from PD and AD, respectively (Table 1, Figure 2A).

In contrast to the shrinkage of the mean PLT volume, the same NDD pathologies are related to the expansion of the mean volume of RBCs (Figure 2A), also known as macrocytosis. Macrocytosis may indicate various medical conditions, including vitamin B12 and folate deficiencies, liver disease, and hypothyroidism [246,247]. A research investigation covering 82 patients with multiple sclerosis (MS) showed that the majority of them had mean RBC volumes higher than those of healthy individuals at the early stage of the disease, and 27% of the MS patients had even abnormally high mean RBC volumes [248].

Our data revealed that the average RBC volume is doubled for ALS patients, and ca. 1.4–1.5 times higher for PD and AD patients, respectively, compared to healthy controls (Figure 2A). This corresponds well to studies that reported enlarged mean RBC volume in other NDDs, and its relation to the development of NDDs and vitamin B12 deficiency ([247] and references therein).

**Figure 3:**
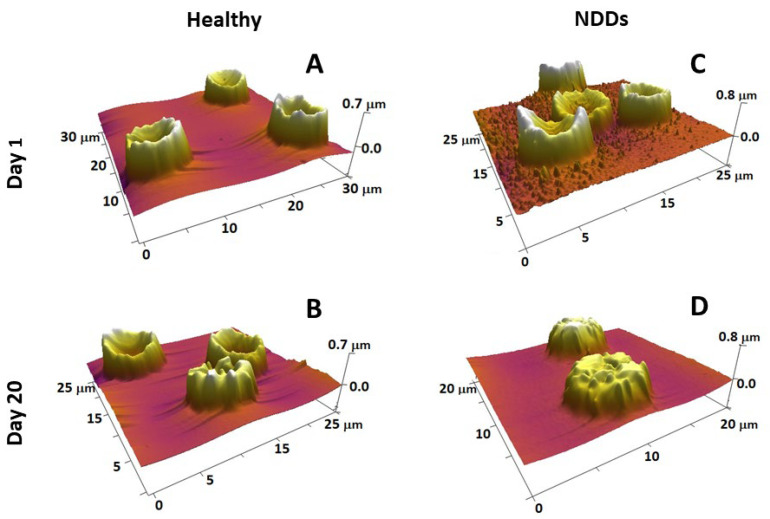
3D AFM images of fresh (**A**,**C**) and 20-day-aged (**B**,**D**) RBCs from healthy (**A**,**B**) and NDD (ALS) (**C**,**D**) donors, detected on smears of the cells on glass support, show that the scanned area is 20–30 µm (given on each image). Images were detected in tapping mode in air with a frequency of 16 kHz and a spring constant of 0.06 N/m using standard silicon nitride (Si3N4) probe tips with a radius <10 nm.

It is to be noted that normal, mature RBCs are characterized by the easily recognizable biconcave discoid shape (ca. 73%), although the presence of small amounts of abnormal-shaped cells (crenated (ca. 21%), spiculated (ca. 6%), and occasionally single spherocite) is not uncommon (Figure 3) [231]. The elevation of the relative proportion of such abnormal-shaped RBCs (poikilocytosis), i.e., the transformation of RBC morphology from normal poikilocytosis to spheroechinocytes, is regarded as a sign of pathological conditions like anemia, hereditary spherocytosis, hereditary elliptocytosis, McLeod syndrome, thalassemia, etc. [241,249]. In vitro studies showed that the decrease in the level of oxygen, i.e., in conditions of hypoxemia/anoxemia, was associated with changes in RBC morphology from discocytes to echinocytes, microspherocytes, and the appearance of ghosts [250].

Our study on the relative share of biconcave, crenated, spiculated, and spherical RBCs in NDDs showed that although the biconcave remained the most abundant shape in ALS and AD, the ratio biconcave:crenated was 2:1, as opposed to the healthy RBSs, where this ratio was 3.2:1 [231]. Most dramatic and specific was the PD pathology characterized by prevailing crenated-shaped RBCs (about 60%) and only about 35% normal-shaped biconcave cells [231]. The morphology of RBCs was found to be significantly affected in AD and PD by high ferritin levels [251]. Pretorius et al. [252] have shown that inflammatory signaling can induce damage to the morphology of RBCs (cell shrinkage and membrane blebbing) and apoptosis (eryptosis) in PD patients.

RBC aging is a complex process of particular scientific and clinical importance. It is a unidirectional physiological event that includes a number of physicochemical changes that regulate their turnover. Detailed knowledge on the transformation of RBC morphology and also on the role of structural and functional proteins in the development of specific morphological intermediate states along the RBCs’ aging path has been gained by exploring AFM [197]. The four most typical cell shapes observed along the aging path are biconcave, crenate, spiculed (echinocytes), and spherocytic [197,231]. Along the course of cell aging, the proportion of discocytes and crenate-shaped cells is reduced at the expense of an increasing proportion of the other two morphological types (spicules and spherocytes) (Figure 3A,B). The reduction is much stronger and much faster in NDD RBCs than in healthy conditions (as seen for ALS in Figure 3C,D). Importantly, the age-induced transformation of RBC morphology followed different pathways in PD, ALS, and AD compared to normal healthy states; for example, the spiculated and spherocytic shapes were the main fractions for PD, ALS, and AD cells at day 20 along the aging process, while the biconcave shape was still the highest fraction in the healthy cells [231]. Furthermore, the contribution of spherocytes to the morphology of PD, ALS, and AD RBCs increased much faster already at an early stage of aging when no spherocytes were present in the healthy cells [231]. The cross-relationship between proteins, such as spectrin, band 4.1 or 4.2, and the cytoplasmic domains of band 3 protein, all involved in the cytoskeleton structure and membrane anchoring, was probed by calorimetry and revealed the stabilizing role of Band 3 cytoplasmic domain on Hb, suggesting that the aging-related morphological changes of RBCs depend mostly on cytoskeleton alterations [197]. We established that NDDs can be differentiated from the normal healthy state based on the variation in the thermodynamic parameters of the unfolding of major RBC proteins [253].

**Figure 4:**
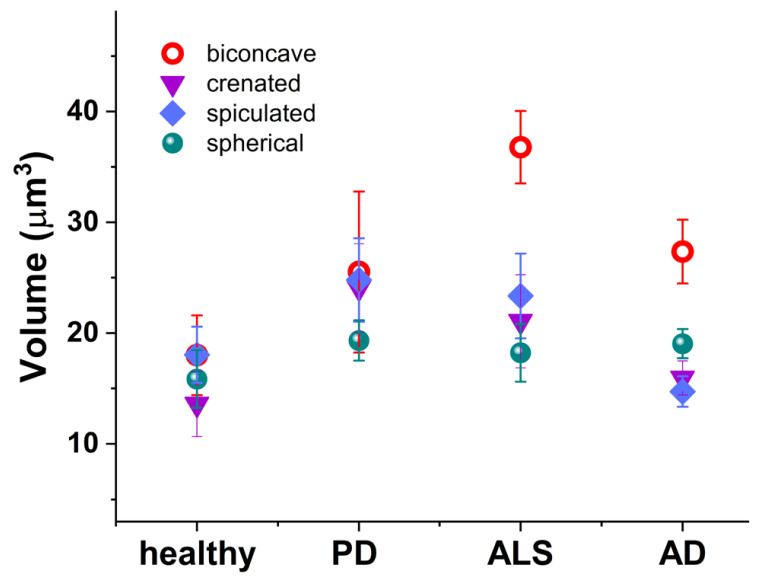
Volume of the RBCs’ morphological types (biconcave (red circle); crenated (violet inverted triangle); spiculated (blue rhombus); and spherical (green shpere)) in healthy individuals, PD, ALS, and AD mean values and SD.

Not only the abundance but also the volume of these abnormally-shaped RBCs undergoes a change in the development of NDDs (Figure 4). The volume of biconcave cells was higher in all NDDs compared to healthy cells; the crenated and spiculated cells were enlarged in PD and ALS, while the spherical ones were relatively unchanged in health and pathological conditions.

**Figure 5:**
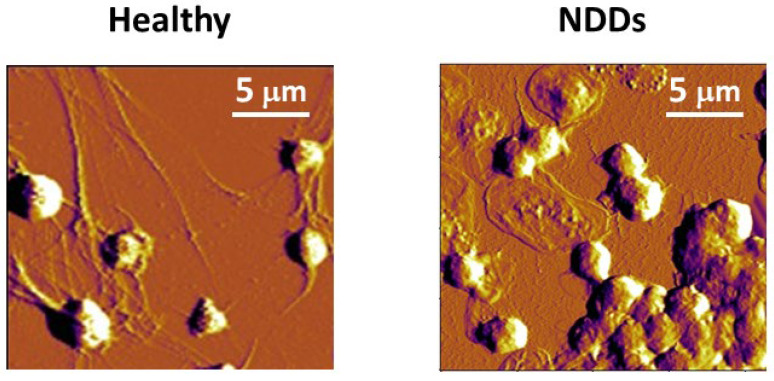
Representative AFM images of platelets from a healthy subject and from a patient with ALS were detected in contact mode using silicon nitride probes with a spring constant of 0.06 N/m, a resonant frequency of 16 kHz, a conical shape, and a nominal tip radius of 8 nm.

Although the volume of RBCs is clearly increased in NDD pathologies, their spreading area is slightly decreased (Figure 2A,B). This decrease correlates very well with the cell membrane stiffening (see the increase in RBCs’ Young’s modulus in Figure 2D). Shrinkage of the spreading area is more significant in PLTs than in RBCs and also correlates perfectly with the increase in Young’s modulus (Figure 2D). The ALS disorder triggers the highest contraction of the spreading area, corresponding to the greatest stiffening of the PLT cell membrane. The reason lies in the inherent ability of PLTs to activate and change their spreading area. The process and degree of PLT activation are formally divided into four stages [150]. While the adhered PLTs from healthy individuals appear in a resting or poorly activated state, in PD they are in an activation stage II or III, in ALS they are in the most advanced stages III and IV (Figure 5, [254]), and finally in AD the PLTs exhibit an activation state II [254].

Along with the cell shape, spreading area, and volume, membrane roughness and stiffness were also found useful for differentiation between healthy persons and patients with acute myocardial infarction [255], type 2 diabetes mellitus (emorheological and atomic force microscopy studies on the experimental clot formations in patients with type 2 diabetes mellitus), hypertension [256], transient ischemic attacks [257], and inherited thrombophilia [258]. We also reported that the PLT membranes in NDDs were significantly stiffer than the control PLTs (Figure 2D, [254]). The greatest membrane rigidification was observed in ALS PLTs and corresponded well with their highest activation and aggregation stages among all NDDs (Figure 2D and Figure 5). It was intriguing to observe that the development of Young’s modulus in PD, ALS, and AD followed a similar trend for both PLTs and RBCs (Figure 2D).

The membrane roughness of RBCs was drastically affected and that of PLTs to a much smaller extent, demonstrating lower values in the studied NDDs than in healthy cells (Figure 2C). The decrease in R_rms_, accompanying the membrane smoothening, was much more pronounced for PD than for ALS and AD RBCs, demonstrating considerably modified cytoskeletal integrity.

**Figure 6:**
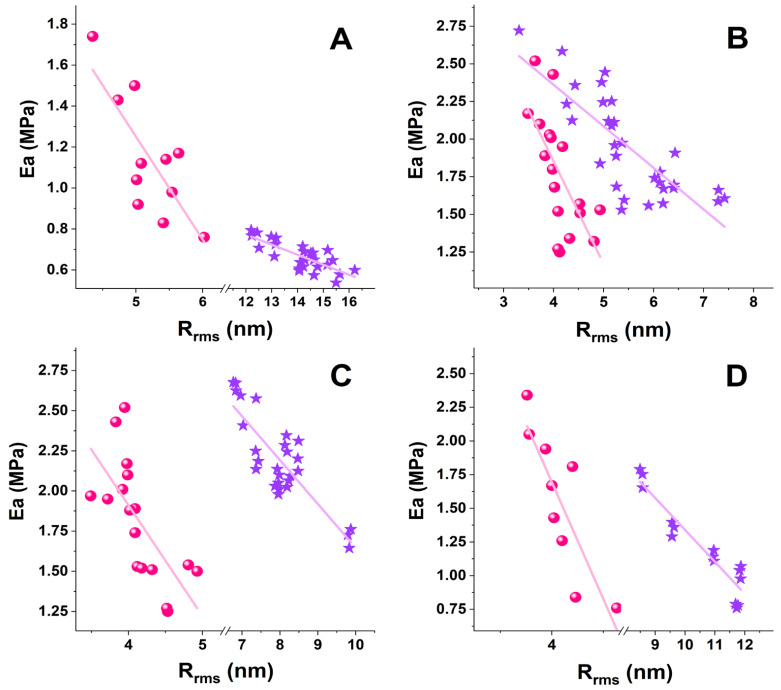
Scatter plots of the stiffness (E_a_), determined by fitting the force-distance curves applying Hertz’s model, vs. the surface roughness (R_rms_, root mean square roughness) derived from the AFM images and force distance curves of RBCs (pink spheres) and PLTs (violet stars) from healthy individuals (**A**) and patients with PD (**B**), ALS (**C**), and AD (**D**).

A relatively strong negative correlative association between R_rms_ and E_a_ of RBCs and PLTs was found for healthy and pathological cells (Figure 6); the correlation coefficient r of the parameter pair E_a_/R_rms_ and a narrow 95% confidence interval (CI) are given in Table 2.

The strongest E_a_/R_rms_ correlation was determined for both RBCs and PLTs in AD, which was supplemented with the narrowest CI for RBCs. The r values were lower for PD and ALS cells than for AD cells, and the narrowest CI was obtained for PD PLTs. The coefficient of determination (r^2^), a measure of the strength of correlation, had values > 0.5 and fell in the range of 0.6–0.86. The r^2^ values showed good results for healthy subjects, PD, and ALS, and very good results for the AD pair of parameters. The correlation coefficients prove that the strength of the relation between the E_a_/R_rms_ pair changes in the same order for the studied disorders (AD (0.72) > ALS (0.48) > PD (0.45) for RBCs and AD (0.86) > ALS (0.7) ≥ PD (0.6) for PLTs).

The E_a_/R_rms_ correlation had the same trend along RBCs’ aging, i.e., a higher correlation coefficient r for AD and a lower correlation coefficient r for PD and ALS compared to healthy cells. A difference was observed for CI, which was wider for 30-day-aged cells than for fresh cells.

Recent experimental evidence suggests that morphological anomalies, including shape alteration and cell swelling, the development of micro-vesicles, proto-spicules, or spicules, a decrease in roughness, and a faster weakening of the membrane-skeleton stability, are associated with the interaction of RBCs with Aβ [259,260]. All of these abnormalities were found to accompany the progression of RBC aging and were accelerated by Aβ [259,260]. Besides, clustering of proteins or lipids on the membrane as a consequence of the interaction of Aβ with cells was observed in the AFM images.

Importantly, previous findings on metabolic RBC disturbances related to AD [261] have been supported by the observed enhancement of the Aβ-dependent effects on RBCs’ morphology by glucose depletion through affecting cytoskeletal integrity [159,260].

Our pilot study on the interaction of PLTs with Aβ has shown similar changes in the morphology (decrease in the area, height, and R_rms_), nanomechanics (increase in E_a_), aggregation, and activation states of PLTs (unpublished data) as observed for PLTs from patients with AD [254]. The AFM studies of Dinarelli et al. [259,260] and our results suggest that the altered morphological and nanomechanical signatures of RBCs and PLTs in AD could be attributed at least in part to the cells’ interaction with Aβ.

The use of AFM and advanced AFM imaging would provide further insight into the blood cells’ biophysical properties in other neurodegenerative disorders, the accumulation of toxic proteinaceous aggregates, and the effect they exert on peripheral blood cell properties.

### 4.4. Nanoscale Structural Features and Dynamics of Amyloidogenic Proteins

AFM and AFM-based techniques have been applied to characterize the aggregation of amyloidogenic proteins—the transformation of misfolded monomers into stable oligomeric intermediates and insoluble fibrils—and the kinetics of amyloid fibril formation.

In situ AFM assessed Aβ aggregation on solid surfaces and revealed the formation of Aβ fibrils dependent on interactions at the hydrophobic substrates/aqueous solutions interface [262]. This study showed a pronounced dependence of the size, shape, and kinetics of Aβ aggregate formation on the physicochemical nature of the surface. Furthermore, Aβ42 aggregation on lipid bilayer surfaces and transformation of Aβ42 monomers to misfolded, aggregation-prone conformations were revealed by time-lapse AFM imaging [263]. AFM combined with time-lapse AFM, high-speed AFM (HS-AFM), and nanoinfrared AFM (nanoIR AFM) provided nanoscale structural details on the secondary structure of the Aβ peptides, the kinetics of fibril formation, their interactions with model lipid membranes, and their dependence on the membrane lipid composition [264]. Visualization of Aβ42 fibril nucleation and fibril elongation were achieved by HS-AFM, as was the growth of straight and spiral fibrils, and moreover, morphological switching between these two morphomers was demonstrated [265]. Study of the oligomer dynamics by means of time-lapse HS-AFM showed a dynamic equilibrium of heptamers with dimers and trimers, suggesting that the higher order of peptide assembly can be blocked by targeting the two lower order types of Aβ structures that have therapeutic significance [266]. A method was developed for the study of amyloids at various stages of their assembly (low molecular weight oligomers, protofibrils, and mature fibers) that allowed nanomechanical mapping of Aβ on a poly-L-lysine (PLL) coated mica substrate using ultrasonic force microscopy (PLL-UFM) and showed the presence of small Aβ42 oligomers even at late stages of fibril assembly [267].

AFM was the biophysical approach used to quantitatively characterize the α-syn aggregation into β-sheet fibrillar structures and the aggregation intermediates on the path-way to α-syn fibril formation [268], which were thought responsible for α-syn toxicity rather than the fibrillar structures [269]. High-resolution AFM and single-molecule force spectroscopy directly showed the formation of protofilaments from the assembly of unfolded monomeric α-syn and determined the smallest elementary unit in the hierarchical assembly of α-syn amyloid fibrils [268]. Moreover, it has been demonstrated that the disease-associated mutations of α-syn generate different amyloid fibril polymorphs compared to the wild type and that a single point mutation can alter the distribution of fibrillar polymorphs in α-syn [270]. These findings indicate that different clinical phenotypes of familial PD could be associated with aggregates with different structures and mechanisms of formation [270].

Recently, a combination of AFM with several other techniques (surface tension measurements, FTIR spectroscopy, and aggregation assays) has been applied to study the self-assembly of β-syn and the role of interfacial effects on the primary nucleation of β-syn [271]. It has been proven that self-assembly of β-syn into amyloid aggregates can occur by homogeneous primary nucleation with a preference for an antiparallel β-sheet arrangement and without the need for an active surface [271].

Importantly, AFM-based comparison of α-syn and Aβ aggregation processes showed significant differences between the two proteins at the early stage of aggregation—mainly monomeric forms of α-syn and oligomeric species of Aβ, while at the late stage of aggregation fibrils and protofibrils were detected for both α-syn and Aβ [272].

### 4.5. AFM versus Other Imaging Techniques

As discussed above, AFM provides valuable information on the morphology, structure, and molecular forces of biomolecules, cells, and tissues. Different AFM operational modes (AFM imaging, AFM-force spectroscopy, nanoindentation) and a number of AFM-based techniques developed in the last decades (HS-AFM [264,265,273,274], time-lapse AFM imaging [275,276], nanoIR AFM [264,268,277,278,279], Tip-Enhanced Raman Spectroscopy (TERS) AFM [280,281,282], ultrasonic force microscopy (UFM) [267], etc.) have shown a number of important applications. AFM combined with analysis of the ultrastructure of RBCs and PLTs, obtained by scanning electron microscopy (SEM) and transmission electron microscopy (TEM), distinguished between diseased and healthy cells [245,283,284].

The main advantages of AFM—sub-nanometer scale resolution for imaging surfaces in both air and in liquid/physiological conditions; recording single molecular events; no sample pretreatments (use of contrast agents and nano-coating with gold or carbon in SEM); probing the response of single cells experiencing deformation; determining the cell adhesion properties; recognition of antigenic sites on the cell membrane surface using a functionalized with antibody AFM probe—have proven the technique useful in various applications.

However, conventional AFM has some disadvantages: a low speed of imaging acquisition that hinders molecular dynamics observations [285] and leads to low-throughput measurement, and a lack of information on the chemical features of the studied specimen. When it comes to the analysis of large surfaces, AFM appears much slower than SEM. AFM can only image a region with a maximum height of 10–20 μm and a maximum scanning area of approximately 150 by 150 μm, which may be a serious factor limiting the clinical prospects of this method. Randomly occurring tip-sample drifts remain a persistent problem in AFM but may only be significant for observing objects with dimensions of a few nanometers. Moreover, distortion of the images of soft samples due to strong tip-sample interactions is a common event. Another artifact known as the “tip-deconvolution effect” can cause an object to be artificially enlarged but can be removed through a process called “deconvolution” using a simple equation and is again relevant for objects with small nanometric dimensions. It should be noted, however, that most of the above-listed AFM artifacts can be avoided by appropriate, in-depth training of AFM operators. Many AFM drawbacks were already overcome by advanced AFM-based techniques. The low speed of imaging acquisition was overcome by HS-AFM, which allowed fast recording of biomolecular dynamics in real time [285,286] and characterization of amyloid peptide and protein aggregation ([287] and refs. from Section 4.4). NanoIR AFM [277,278] and TERS AFM [280,281] provided chemical and structural information in real-time.

A novel construction of a parallel instrument combining many miniature AFMs that operate in parallel has been developed for high-throughput sub-nanometer imaging [288]. This parallel AFM would allow simultaneous recordings of different properties of a large number of cells and is an important step toward one of the most ambitious challenges—clinical application of AFM. Alternatively, another strategy to increase the imaging throughput is based on simultaneous multi-cantilever operation in parallel that explores an array of active cantilevers with embedded piezoresistive sensors and thermomechanical actuators, in contrast to the single passive cantilever used in conventional AFM [289].

Furthermore, microfluidic constrictions with various geometries built to study cellular mechanics at a single cell level ([290,291,292] and references therein) provided valuable information on RBC membrane disorders (deformability, membrane surface area, surface-to-volume ratio). These constructions were able to discriminate changes in the surface area from changes in the deformability of RBC membranes [291], adult cells from early-stage cells [293], and disease cells from healthy ones [290]. Importantly, Faustino et al. [294,295] using a microchannel implementing a hyperbolic constriction assessed the deformability of pathological cells and succeeded in differentiating RBCs in contact with tumoral cells from healthy RBCs [294] and healthy RBCs from cells derived from end-stage kidney disease patients [295]. Measurements of the recovery time of discocyte shape have also been performed in microfluidics [296,297,298]. A pilot study of the mechanical responses of RBCs from a few patients with hereditary spherocytosis and sickle cell anemia, artificially rigidified in a microfluidic constriction, proved the potential of this approach for diagnostic applications [292]. The advantages of microfluidic systems in modeling neurodegenerative diseases and their capability to integrate components into “new generation” small lab-on-a-chip devices (brain-on-a-chip microfluidic culture platforms for AD and PD pathologies and spinal-cord-on-a-chip methods for ALS) were discussed in the review of Osaki et al. [299].

Finally, it is expected that the use of AFM and advanced AFM imaging will provide further insight into the blood cells’ biophysical properties in other neurodegenerative disorders, the accumulation of toxic proteinaceous aggregates, and the effect they exert on peripheral blood cell properties.

## 5. Conclusions

AFM is a promising instrument for identifying the development of neurodegenerative pathologies and their differentiation. Both the nanotopographic imaging and the force-distance curves provide valuable information on the morphological and nanomechanical cell features that can be helpful in the diagnostics of NDDs. In particular, the morphology, membrane surface roughness, and nanomechanics of PLTs and RBCs can be used to distinguish pathological cells from normal healthy cells, as well as ALS, PD, and AD from each other. The strong correlations between morphological and mechanical measures of PLTs and RBCs for each of the pathologies studied are striking.

Common hallmarks of PLTs and RBCs have been identified that provide a set of biophysical markers for the diagnosis of the three pathologies: volume, height, spreading area, membrane roughness, and Young’s modulus of the two types of blood cells; PLT level of activation and granule development and release; aging patterns of RBC morphological and mechanical parameters; and level of poikilocytosis. Among those biophysical markers, the strongest decrease in surface roughness and prevailing crenate-shaped RBCs are specific for PD. Modifications of membrane roughness can be used to reveal the mechanisms underlying the development of specific pathologies.

Our findings strongly support the potential of AFM to discriminate neurodegenerative disorder-based morphological and nanomechanical signatures of peripheral blood cells; the next challenge is the validation of AFM application in medical diagnostics.

## Figures and Tables

**Table 1:** Platelet volume (mean values and SD) for healthy individuals and patients with NDDs determined from the AFM images analyzed using Gwyddion-2.57 and IgorPro 6.37 software, and NDDs values given in percentage of the value of healthy platelets.

Condition	Mean PLTs Volume, µm^3^	% of Healthy PLTs Volume	% of Shrinkage Relative to Healthy PLTs Volume
Healthy	3.20 ± 1.28	-	-
PD	1.48 ± 0.34	46.3	55.7%
ALS	1.13 ± 0.34	35.3	64.7%
AD	2.19 ± 0.55	68.4	31.6%

**Table 2:** Pearson’s correlation coefficient (r) for the pair of nanomechanical (stiffness (E_a_)) and morphological (surface roughness (R_rms_)) parameters of RBCs and PLTs derived from healthy and NDD subjects. Confidential interval (CI), lower and upper limits.

	RBCs			PLTs		
Subject	E_a_/R_rms_	CI		E_a_/R_rms_	CI	
	r	Lower Limit	Upper Limit	r	Lower Limit	Upper Limit
Healthy	−0.78	−0.935	−0.373	−0.75	−0.8794	−0.5175
PD	−0.67	−0.875	−0.261	−0.78	−0.8887	−0.5882
ALS	−0.69	−0.879	−0.313	−0.84	−0.9248	−0.6757
AD	−0.85	−0.972	−0.562	−0.93	−0.9769	−0.7978
